# 4-methylumbelliferone (4-MU) enhances drought tolerance of apple by regulating rhizosphere microbial diversity and root architecture

**DOI:** 10.1093/hr/uhad099

**Published:** 2023-05-19

**Authors:** Dehui Zhang, Jieqiang He, Pengda Cheng, Yutian Zhang, Abid Khan, Shicong Wang, Zhongxing Li, Shuang Zhao, Xiangqiang Zhan, Fengwang Ma, Xuewei Li, Qingmei Guan

**Affiliations:** State Key Laboratory of Crop Stress Biology for Arid Areas/Shaanxi Key Laboratory of Apple, College of Horticulture, Northwest A&F University, Yangling 712100, China; College of Life Science, Northwest A&F University, Yangling 712100, China; State Key Laboratory of Crop Stress Biology for Arid Areas/Shaanxi Key Laboratory of Apple, College of Horticulture, Northwest A&F University, Yangling 712100, China; State Key Laboratory of Crop Stress Biology for Arid Areas/Shaanxi Key Laboratory of Apple, College of Horticulture, Northwest A&F University, Yangling 712100, China; State Key Laboratory of Crop Stress Biology for Arid Areas/Shaanxi Key Laboratory of Apple, College of Horticulture, Northwest A&F University, Yangling 712100, China; Department of Horticulture, The University of Haripur, Haripur 22620, Pakistan; State Key Laboratory of Crop Stress Biology for Arid Areas/Shaanxi Key Laboratory of Apple, College of Horticulture, Northwest A&F University, Yangling 712100, China; State Key Laboratory of Crop Stress Biology for Arid Areas/Shaanxi Key Laboratory of Apple, College of Horticulture, Northwest A&F University, Yangling 712100, China; State Key Laboratory of Crop Stress Biology for Arid Areas/Shaanxi Key Laboratory of Apple, College of Horticulture, Northwest A&F University, Yangling 712100, China; State Key Laboratory of Crop Stress Biology for Arid Areas/Shaanxi Key Laboratory of Apple, College of Horticulture, Northwest A&F University, Yangling 712100, China; State Key Laboratory of Crop Stress Biology for Arid Areas/Shaanxi Key Laboratory of Apple, College of Horticulture, Northwest A&F University, Yangling 712100, China; State Key Laboratory of Crop Stress Biology for Arid Areas/Shaanxi Key Laboratory of Apple, College of Horticulture, Northwest A&F University, Yangling 712100, China; State Key Laboratory of Crop Stress Biology for Arid Areas/Shaanxi Key Laboratory of Apple, College of Horticulture, Northwest A&F University, Yangling 712100, China

## Abstract

The dwarfing rootstocks-mediated high-density apple orchard is becoming the main practice management. Currently, dwarfing rootstocks are widely used worldwide, but their shallow root system and drought sensitivity necessitate high irrigation requirements. Here, the root transcriptome and metabolome of dwarfing (M9-T337, a drought-sensitive rootstock) and vigorous rootstocks (*Malus sieversii*, a drought-tolerant species, is commonly used as a rootstock) showed that a coumarin derivative, 4-Methylumbelliferon (4-MU), was found to accumulate significantly in the roots of vigorous rootstock under drought condition. When exogenous 4-MU was applied to the roots of dwarfing rootstock under drought treatment, the plants displayed increased root biomass, higher root-to-shoot ratio, greater photosynthesis, and elevated water use efficiency. In addition, diversity and structure analysis of the rhizosphere soil microbial community demonstrated that 4-MU treatment increased the relative abundance of putatively beneficial bacteria and fungi. Of these, *Pseudomonas*, *Bacillus*, *Streptomyces*, and *Chryseolinea* bacterial strains and *Acremonium*, *Trichoderma*, and *Phoma* fungal strains known for root growth, or systemic resistance against drought stress, were significantly accumulated in the roots of dwarfing rootstock after 4-MU treatment under drought stress condition. Taken together, we identified a promising compound—4-MU, as a useful tool, to strengthen the drought tolerance of apple dwarfing rootstock.

## Teaser

We identified a promising compound—4-MU, as a useful tool, to facilitate the drought tolerance of apple dwarfing rootstock by regulating rhizosphere microbial diversity and root architecture.

## Introduction

As global surface temperature increase, so does the likelihood of extreme weather, including drought stress. Numerous studies have demonstrated that drought stress influences the crop quality as well as their growth, survival and yield [1]. Because of the flavor and nutritional value, apple is an important fruit crop with a long history of cultivation [2]. However, the low annual rainfall and its uneven seasonal distribution in apple-producing region often cause seasonal drought, restricting the sustainability of the apple industry [3].

Grafting improves apple performance by using rootstocks to enhance scion yield, quality, and resilience to biotic and abiotic stresses [4]. Rootstocks provide the plant with nutrients and water uptake and facilitate drought tolerance through a variety of mechanisms, such as root biomass adjustments (root-to-shoot ratio), anatomical changes, and physiological acclimations, which allow the plants to avoid and tolerate environmental stresses [5]. *Malus sieversii*, a species with deep roots, is extremely tolerant to drought stress and commonly used as a vigorous rootstock [6]. However, as high-density planting systems with dwarfing rootstocks have been wildly accepted [7], it’s very difficult for *M. sieversii* to be promoted in the modern apple orchards. The M9-T337 dwarfing rootstock is widely used worldwide due to its ability to promote early fruiting and high scion yield since its initial identification [8]. However, due to M9-T337's shallow root system, it is prone to suffer from drought stress. Therefore, exploring an economical and high-efficient way to enhance the root system of dwarfing rootstock will benefit the apple industry.

The plant kingdom contains various types of secondary metabolites with heterogeneous structural characteristics, activities, and properties [9]. Lots of these secondary metabolites have crucial roles in controlling how plants react to biotic and abiotic stress, signal transduction, oxidative stress, nutrient uptake, and development [10]. The metabolomics, defined as the bridge between the phenotype or genotype of a plant and the “readout” of the physiological status, has been an excellent way to explore the diversity of metabolism as well as the molecular mechanisms by which plants respond and alleviate various stressors [11]. Coumarin is an important secondary metabolite discovered originally from *Coumarouna odorata* in 1820 [12]. A number of studies revealed the rapid increase of coumarins upon environmental stresses, such as pathogen infection, iron deficiency, salinity stress, cold stress, and drought stress [13–17], which have emerged as an adaptive response to mitigate various stresses. A key regulator of coumarin availability and biological activity in plants is glycosylation. Once synthesized, coumarins are present in the plant cell as glycosylated (glycoside: e.g. scopolin, fraxin, sideretin glycosides, or esculin) and deglycosylated (aglycone: e.g. scopoletin, fraxetin, sideretin, or esculetin) forms [18]. In plants, the glycosylation of coumarins occurs in the cytoplasm by UDP-glycosyltransferases (UGTs) and additionally, coumarins in their glycoside form are easier to transport and store in vacuoles [18]. When plants are subjected to environmental stress, coumarin glycosides stored in vacuoles can be deglycosylated into bioactive aglycones through beta-glucosidase enzymes and then are released into the rhizosphere [19, 20].

Due to the crucial roles that rhizosphere microbiomes play in plant growth and biotic or abiotic stress response [21], the collective genome of soil microbial community has been used to reveal the complex interactions between plants and their rhizosphere microbes [22]. Recent studies have found that plant metabolites can influence the microbial community or maintain the plant's healthy growth [23]. A previous study demonstrated that plants communicate via lipids, phenolic acids, organic acids, and amino acids with beneficial bacteria and fungi to stabilize communities that mitigate the grazing stress in typical steppe ecosystems [24]. In another study, glutamic acid directly reshaped the composition of the microbiome community to protect plants from pathogens [25]. Recent studies have uncovered a crucial role of coumarins in iron acquisition through reduction and chelation [26]. Interestingly, scopoletin (a coumarin derivative) selectively accumulates rhizobacteria *Pseudomonas*, whereas inhibits fungal pathogens *Fusarium oxysporum* and *Verticillium dahliae,* thereby triggering an induced systemic resistance and iron uptake, as shown by analysis of microbiota assemblies of *myb72* and *f6’h1* mutants in *Arabidopsis* [27, 28]. Additionally, plant root microbes are associated with the drought resistance of host plants, such as *Pseudomonas putida* [29], *Pseudomonas chlororaphis* [30], *Bacillus thuringiensis* [31], *Streptomyces rimosus* [32], *Acremonium coenophialum* [33], *Trichoderma hamatum* [34], *Phoma medicaginis* [35], and arbuscular mycorrhizal fungi [36]. However, in apple plants it remains unelucidated whether apple plants reshape the rhizosphere microbiota that alleviate drought stress by altering root metabolites.

In the current study, we characterized root transcriptional and metabolic profiling of dwarfing and vigorous rootstocks in response to drought, and found that 4-Methylumbelliferone (4-MU, a coumarin derivative) could be significantly induced by drought stress in vigorous rootstock. Exogenous inoculation of 4-MU to the roots of dwarfing rootstock enhanced its drought tolerance, which might be partially due to its regulation of root growth and correlated with a putatively beneficial shift in microbiome composition. Our results provided vital information about metabolic reprogramming and microbial community reshaping of apple rootstocks under drought stress, as well as a chemical compound to strengthen drought tolerance of apple dwarfing rootstocks.

## Results

### Transcriptomic analysis of vigorous and dwarfing rootstocks in response to drought stress

Previous studies showed that *M. sieversii* is more tolerant to drought stress than M9-T337 [37, 38]. After long-term drought stress, we found that *M. sieversii* exhibited less shoot dry weight than M9-T337, resulting in a higher root-to-shoot ratio and indicating greater drought tolerance compared to M9-T337, despite being smaller in plant height and stem diameter (Supplemental Figures 1A-E). In addition, *M. sieversii* had a higher photosynthetic rate (Pn), stomatal conductance (Gs), intercellular CO_2_ concentration (Ci), and transpiration rate (Tr) (Supplemental Figures 1G-J), further suggesting the better performance of *M. sieversii* under drought stress.

**Figure 1:**
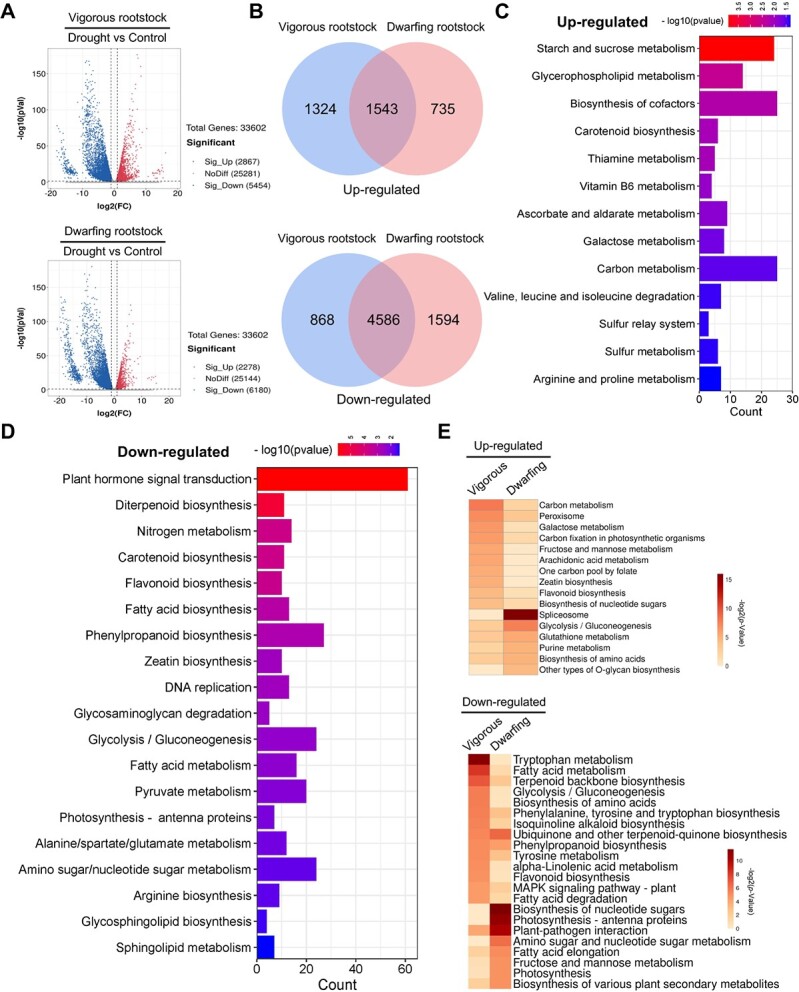
Transcriptome profiling analysis of roots in vigorous and dwarfing rootstock in response to drought stress. (A) Volcano plots of drought-responsive genes in vigorous or dwarfing rootstock. (B) Venn diagrams of up- and down-regulated genes in vigorous or dwarfing rootstock in response to drought condition. (C) KEGG pathway assignment of 1543 up-regulated genes in (B). (D) KEGG pathway assignment of 4586 down-regulated genes in (B). (E) KEGG pathway assignment of specifically impacted genes in vigorous (1324 up- or 868 down-regulated genes in B) and dwarfing (735 up- or 1594 down-regulated genes in B) rootstocks by drought. The heat map presents statistical significance (log2-transformed *p-*value) of KEGG pathway term. *Malus sieversii* and M9-T337 represent vigorous and dwarfing rootstock, respectively.

To explore the difference in drought tolerance between vigorous and dwarfing rootstocks, we detected the root transcriptome of *M. sieversii* and M9-T337 under control and drought conditions. The data we obtained suggested that the quality of RNA-seq was high and reliable (Supplemental Table S1). In response to drought stress, we identified 8321 (2867 up-regulated and 5454 down-regulated), and 8458 (2278 up-regulated and 6180 down-regulated) differentially expressed genes (DEGs) from vigorous and dwarfing rootstocks, respectively (Figures 1A and B; Supplemental Tables S2 and S3). As shown in Figure 1B and Supplemental Table S4, a total of 1543 up-regulated and 4586 down-regulated overlapped genes were found in both vigorous and dwarfing rootstocks. The KEGG pathway enrichment analysis indicated that the 1543 up-regulated genes were significantly enriched in starch and sucrose metabolism, glycerophospholipid metabolism, biosynthesis of cofactors and so on (Figure 1C; Supplemental Table S5), while 4586 down-regulated genes were enriched in plant hormone signal transduction, diterpenoid biosynthesis, nitrogen metabolism as well as carotenoid biosynthesis (Figure 1D; Supplemental Table S6).

Venn diagrams also showed that the vigorous rootstock had 1324 up-regulated and 868 down-regulated DEGs, while the dwarfing rootstock had 735 up-regulated and 1594 down-regulated DEGs (Figure 1B; Supplemental Tables S7 and S8). We applied the KEGG enrichment analysis among the DEGs to elucidate the different biological process in the two rootstocks in response to drought. The results showed that in vigorous rootstock, the 1324 up-regulated DEGs were enriched in pathways of carbon metabolism, galactose metabolism, carbon fixation in photosynthetic organisms and so on. While the 735 down-regulated DEGs were enriched in tryptophan metabolism, fatty acid metabolism, alpha-Linolenic acid metabolism and so on (Figure 1E; Supplemental Tables S9 and S10). Similarly, in dwarfing rootstock, the 868 up-regulated DEGs were involved in spliceosome, glutathione metabolism, glycolysis/gluconeogenesis and so on. While the 1594 down-regulated DEGs were involved in biosynthesis of nucleotide sugars, photosynthesis-antenna proteins, biosynthesis of various plant secondary metabolites and so on (Figure 1E; Supplemental Tables S9 and S10).

Taken together, the above results suggest that the vigorous and dwarfing rootstocks have evolved a series of adaptations to cope with drought stress through common and unique pathways at transcriptomic level.

### Root metabolomic analysis of vigorous and dwarfing rootstocks in response to drought stress

**Figure 2:**
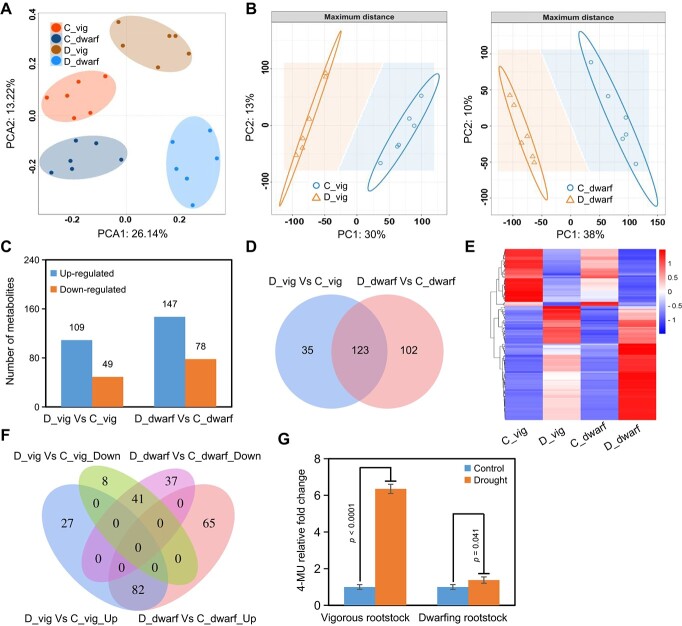
Changes in metabolites of vigorous and dwarfing rootstocks in response to drought stress. (A) Principal component analysis (PCA) of metabolomics data of vigorous and dwarfing rootstocks in response to drought stress. (B) Partial least square discriminant analysis (PLS-DA) of dynamic metabolites in vigorous or dwarfing rootstock in response to drought stress. (C, D) Number (C) and Venn diagrams (D) of drought-responsive metabolites in vigorous and dwarfing rootstocks. (E) Heat map of 123 co-regulated metabolites related to drought stress in vigorous and dwarfing rootstocks. (F) The shared and unique metabolites between vigorous and dwarfing rootstocks in response to drought stress. (G) Relative fold change of 4-MU in vigorous and dwarfing rootstocks under control and drought conditions. D_vig: vigorous rootstock under drought treatment, C_vig: vigorous rootstock under control condition, D_dwarf: dwarfing rootstock under drought treatment, C_dwarf: dwarfing rootstock under control condition. *Malus sieversii* and M9-T337 represent vigorous and dwarfing rootstock, respectively. *p* values from Student’s two-tailed *t*-test.

To investigate the root metabolite changes in response to drought stress, we performed metabolomic profiling for vigorous and dwarfing rootstocks under control and drought conditions. A total of 953 metabolites were identified in all samples, including primary and secondary metabolites such as amino acids, organic acids, sugar alcohols and so on (Supplemental Figure 2; Supplemental Table S11). As shown in Figure 2A, the principal component analysis (PCA) suggested that the drought treatment groups were clearly separated from the control group along the PCA1 that explained 26.14% of total variation; while the separation between vigorous and dwarfing rootstock could be observed by PCA2 explaining 13.22% variation, suggesting a good correlation among the biological replicates.

In order to identify the drought-responsive metabolites (DRMs) in the rootstocks, partial least squares-discriminant analysis (PLS-DA) was performed to compare the metabolome data to maximize the separation between control and drought groups. The PLS-DA score plot showed that drought treatment explained 30% and 38% of the total variation in vigorous and dwarfing rootstock, respectively (Figure 2B). Based on the screening criteria (a fold-change threshold ≥2, *t*-test p ≤ 0.05, and VIP ≥ 1), 158 and 225 drought-responsive metabolites were subsequently isolated, with 109 up-regulated and 49 down-regulated metabolites in vigorous rootstock, and 147 up-regulated and 78 down-regulated metabolites in dwarfing rootstock, respectively (Figures 2C and D; Supplemental Tables S12 and S13). The heatmaps of DRMs between these two rootstocks clearly showed how their patterns changed in response to drought stress (Supplemental Figure 3). As shown in Figure 2D, the two rootstocks under drought shared 123 commonly expressed metabolites which were further divided into two groups (Figure 2E and Supplemental Table S14). Common metabolites in group I including flavonoids, amino acids, sugars and sugar alcohols, organic acids and derivatives, benzenoids, and indoles and derivatives were highly accumulated under drought treatment (Supplemental Table S14). Conversely, common metabolites in group II including benzenoids, organic acids, organooxygen compounds, and lipids and lipid-like molecules were reduced under drought stress (Supplemental Table S14). These metabolites likely account for the typical strategy of the apple rootstock to cope with drought stress condition. Additionally, the Venn diagram revealed that 35 DRMs in vigorous rootstock and 102 DRMs in dwarfing rootstock were genotype-specific (Figure 2F). By further intersection analysis, 27 metabolites exhibited significant increase in response to drought treatment in the vigorous rootstock, including organic acids and derivatives, organ heterocyclic compounds, and coumarins and derivatives (coumarin, 4-Methylumbelliferone, 4-Methylumbelliferyl-D-glucopyranoside) (Supplemental Figure 4; Supplemental Table S15). In addition, 65 metabolites were accumulated significantly in dwarfing rootstock in response to drought, including organic acids and derivatives, sugars, and benzenoids (Supplemental Table S16). Additionally, 8 and 37 metabolites inhibited by drought stress were also found in the two rootstocks, respectively (Figure 2F, Supplemental Tables S15 and S16). Possible explanations for the different response of the two rootstocks to drought treatment might be changed metabolites.

Notably, three metabolites involved in stress tolerance, threonic acid [39, 40], ursolic acid [41], and piperidine [42, 43], were significantly accumulated in vigorous rootstock but reduced obviously in dwarfing rootstock under drought condition (Supplemental Tables S15 and S16). In addition, we found that 4-Methylumbelliferone (4-MU), which could be transformed into 4-Methylumbelliferyl-D-glucopyranoside (4-MUG) by UDP-glycosyltransferases (UGTs) catalysis [44], significantly accumulated in vigorous rootstock under drought; however, it was slightly responsive to drought in dwarfing rootstock (Figure 2G; Supplemental Tables S15 and S16), indicating that 4-MU may play a role in apple drought tolerance.

### Gene expression patterns associated with coumarin biosynthesis pathway

**Figure 3:**
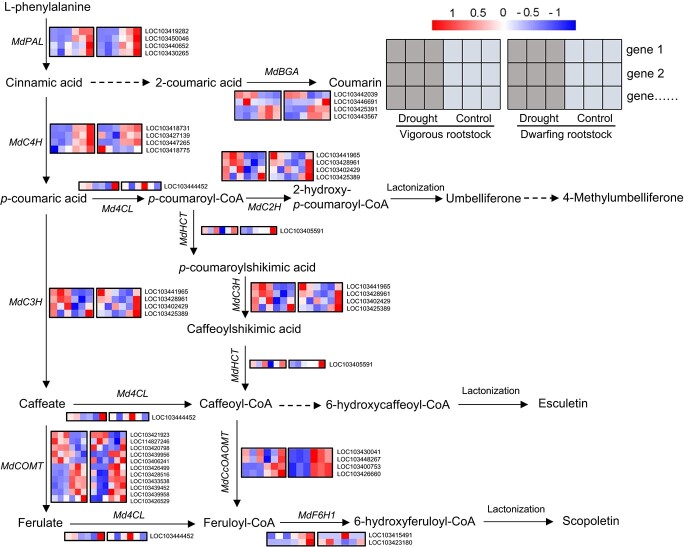
Modulation of coumarin biosynthetic pathway under drought stress in vigorous and dwarfing rootstocks. Red and blue correspond to high and low expression levels, respectively. *Malus sieversii* and M9-T337 represent vigorous and dwarfing rootstock, respectively.

The accumulation of coumarin, 4-MU, and 4-MUG was observed in the roots of vigorous rootstock but not in dwarfing rootstock, highlighting the significance of coumarin biosynthesis. The coumarin biosynthetic pathway is a branch of phenylpropanoid metabolism pathway. Phenylalanine is catalyzed into *p*-coumaric acid by phenylalanine ammonia-lyase (PAL) and cinnamate-4-hydroxylase (C4H) and the *p*-coumaric acid is situated at the crossover point of this branch, which is further converted to *p*-coumaroyl-CoA under catalysis of C4H and 4-coumarate-CoA ligase (4CL). Then, the *p*-coumaroyl-CoA is catalyzed by *p*-coumaroyl CoA 2-hydroxylase (C2H), hydroxy cinnamoyl transferase (HCT), *P*-coumarate 3-hydroxylase (C3H), caffeoyl-CoA O-methyltransferase (CcOAOMT), feruloyl-CoA 6-hydroxylase 1 (F6H1), and lactonization to form umbelliferone, esculetin, and scopolin. The *p*-coumaric acid can also be catalyzed by C3H and caffeic acid O-methyltransferase (COMT) to form caffeate or ferulate which is further converted to esculetin or scopolin. In addition, the conversion of 2-coumaric acid to coumarin is catalyzed by *β*-glucosidase (BGA) (Figure 3) [15, 45].

We also examined the expression pattern of genes involved in coumarin biosynthesis pathway under drought conditions in the two genotypes, including *LOC10344203*9, *LOC103446691*, *LOC103441965*, *LOC103428961*, *LOC103402429*, *LOC103425389, LOC103439956*, *LOC103420798*, *LOC114827246*, *LOC103421923*, and *LOC103406241*, which are the homologous genes of *BGA*, *C3H, C2H,* and *COMT* (Supplemental Figure 5). Compared with the expression profiling of coumarin biosynthetic genes under control condition, almost all the genes we tested had no significant difference in response to drought, except for the *MdBGA*, *MdC2H*, and *MdCOMT*. To verify the expression of the candidate genes, we carried out RT-qPCR analysis. As shown in Supplemental Figure 5, average expression levels of *MdBGA*, *MdC2H*, and *MdCOMT* were significantly increased in vigorous rootstock under drought condition, but decreased or unchanged in dwarfing rootstock.

### Exogenous 4-MU enhances drought tolerance of dwarfing rootstock

**Figure 4:**
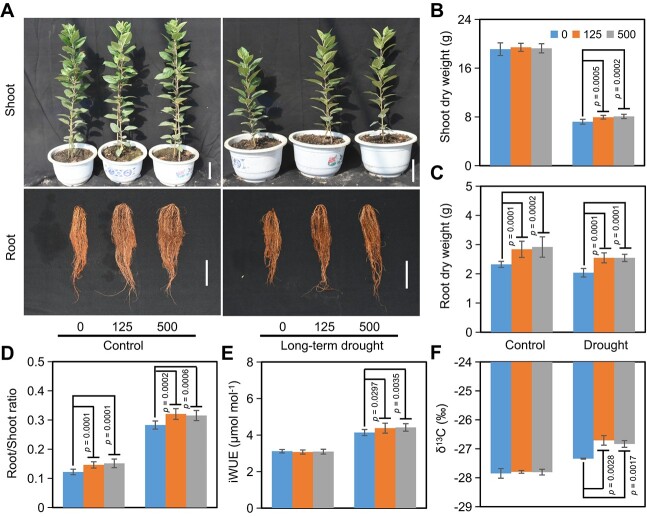
4-MU promotes the drought tolerance of dwarfing rootstock. (A) Effect of exogenous 4-MU (125 and 500 μM) on long-term drought stress response. (B–D) Shoot dry weight (B), Root dry weight (C), and Root/shoot ratio (D) under control and long-term drought conditions. (E) The changes in instantaneous WUE (iWUE) in response to drought stress. (F) The changes in carbon isotopic composition (δ^13^C) in response to drought stress. M9-T337 represents dwarfing rootstock. Error bars indicate standard deviation (n = 10). *p* values from Student’s two-tailed *t*-test.

To examine whether 4-MU played a role in drought tolerance of apple dwarfing rootstock, we determined the plant growth by exogenous application of 125 and 500 μM 4-MU under long-term drought condition. It was obvious that drought stress exerted severe influences on the plant’s growth (Figure 4A). Under control condition, in response to various 4-MU concentrations, there was no difference in the aerial parts including plant height, stem diameter, and shoot dry weight (Figures 4A and B; Supplemental Figure 6A). However, the root dry weight was significantly increased in the 4-MU-treated plants under control condition. Consistently, root-to-shoot ratio calculated was higher in the 4-MU-treated plants under control condition, compared with the control plants (Figures 4C and D). After long-term drought treatment, the 4-MU-treated plants exhibited greater plant height, thicker stem diameter, more shoot and root dry weight as well as higher root-to-shoot ratio, as compared to plants without 4-MU treatment under drought stress (Figures 4A-D; Supplemental Figure 6A). We also measured the photosynthetic parameters, including Pn, Gs, Ci, and Tr in dwarfing rootstock in response to long-term drought stress. Our results showed that the photosynthetic parameters of the drought-treated plants decreased significantly, but under drought condition all the photosynthetic parameters of 4-MU-treated M9-T337 plants were higher than those of non-treated plants (Supplemental Figure 6B). These results suggested that 4-MU can significantly facilitate performance of apple rootstocks under drought. In addition, we investigated the relative water content (RWC) of apple leaves in response to long-term drought condition. The RWC were significantly decreased by the drought stress. However, the 4-MU subsequently alleviated this response in dwarfing rootstock (Supplemental Figure 6C).

Improving water use efficiency (WUE) is an adaptive physiological strategy to respond the drought stress [46]. To examine the effect of 4-MU on the WUE of dwarfing rootstock under drought, we first determined the instantaneous water use efficiency (iWUE) under control and long-term drought conditions. As shown in Figure 4E, iWUE was higher in 4-MU-treated plants as compared to the non-treated plants under drought condition. As an indicator of the physiological and environmental properties of plants, carbon isotopic composition (δ^13^C) can help predict the plants' ability to fix long-term carbon [47]. Since δ^13^C is positively correlated with WUE, δ^13^C can be used to evaluate whole-plant WUE [48]. We therefore investigated δ^13^C under control and long-term drought conditions. Under long-term drought conditions, 4-MU-treated plants exhibited higher δ^13^C than the untreated plants (Figure 4F), further suggesting that 4-MU can improve WUE of apple dwarfing rootstock under drought. In addition, there was no difference in δ^13^C as well as iWUE between 4-MU-treated and non-treated dwarfing rootstock under control condition.

### The transcriptomic analysis of 4-MU-treated dwarfing rootstock under drought stress

**Figure 5:**
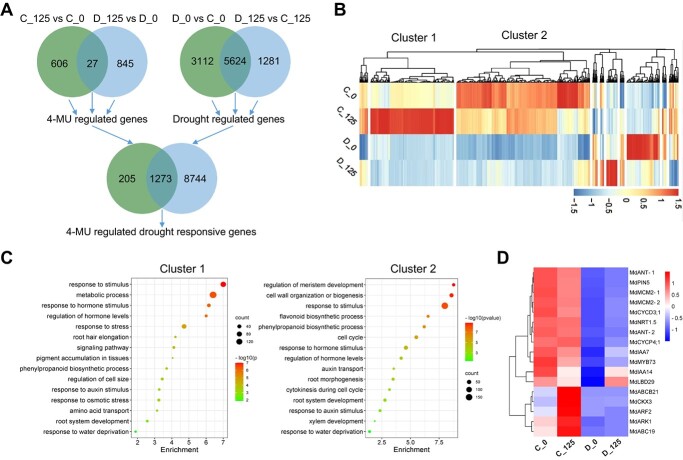
Comparison of differentially expressed genes (DEGs) in dwarfing rootstock in response to 4-MU and drought treatment. (A) Venn diagram showing the overlap of DEGs regulated both by 4-MU and drought stress. (B) Clustering analysis of 4-MU regulated drought responsive genes. (C) Go analysis of genes in cluster1 and cluster 2 shown in B. (D) Heat map of selected genes related to root development shown in C. C_0: M9-T337 under control condition, C_125: M9-T337 under 125 μM 4-MU treatment, D_0: M9-T337 under drought treatment, D_125: M9-T337 under 125 μM 4-MU and drought treatment. M9-T337 represents dwarfing rootstock.

To further explore the biological involvement of 4-MU in response to drought stress, we performed RNA-seq analysis using the M9-T337 roots with 0 and 125 μM (the effect of 125 μM was consistent with that of 500 μM) of 4-MU treatment under control and drought conditions. The results revealed that there were 633 and 872 4-MU-responsive genes in dwarfing rootstock under control and drought conditions, respectively (Supplemental Figure 7; Supplemental Tables S17 and S18). To explore the drought responsive genes, We compared RNA-seq data between plants under drought stress and those under control condition (D_0 vs C_0), as well as between 4-MU treated plants under drought stress and 4-MU treated plants under control condition (D_125 vs C_125) (Supplemental Figure 7; Supplemental Tables S19 and S20). We identified a total of 1273 genes that were regulated by both 4-MU and drought stress (Figure 5A; Supplemental Table S21). Cluster analysis grouped these 1273 4-MU regulated drought responsive genes into several groups (Figure 5B; Supplemental Table S22). We performed gene ontology (Go) analysis of the first two groups (cluster 1 and cluster 2) with the largest number of genes to explore the biological processes. Most notably, high proportions of genes in the two clusters were enriched in root development, response to hormone, or cell cycle (Figure 5C; Supplemental Table S23). Based on the result, the heatmap was used to analyze DEGs involved in response to stress, root development, response to auxin, and cell cycle (Figure 5D).

**Figure 6:**
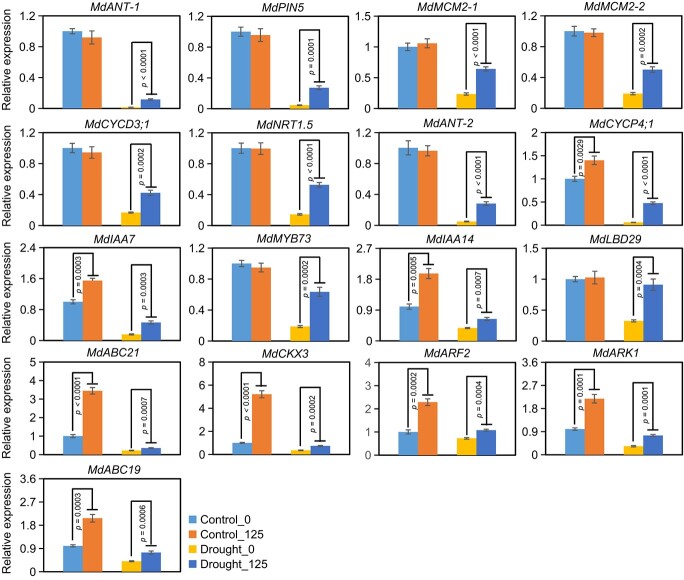
The relative expression of root branching-related genes in dwarfing rootstocks in response to drought stress and 4-MU treatment. Control_0: M9-T337 under control condition, Control_125: M9-T337 under 125 μM 4-MU treatment, Drought_0: M9-T337 under drought treatment, Drought_125: M9-T337 under 125 μM 4-MU and drought treatment. M9-T337 represents dwarfing rootstock. *p* values from Student’s two-tailed *t*-test.

We selected 20 genes and applied RT-qPCR analysis to confirm the reliability of RNA-seq data. Results showed that expression of 17 genes out of 20 genes was confirmed, indicating the reliability of RNA-seq data. Specifically, the expression of *MdIAA14*, *MdABC21*, *MdCKX3*, *MdARF2*, *MdARK1*, and *MdABC19* was higher in 4-MU-treated plants than that in non-treated plants under control condition, and all of the genes were up-regulated by 4-MU under drought condition (Figure 6). These results implied that 4-MU may positively regulate drought tolerance of apple dwarfing rootstock by modulating complex biological processes involved in root development, cell cycle, auxin transport, and auxin or cytokinesis signal transduction.

### Rhizosphere microbiota composition around dwarfing rootstock under control and drought conditions

Since root-exuded coumarins were identified as novel players in communication between plants and soil microbiome [17], so we are interested in investigating whether 4-MU, as a coumarin derivative, could alter the rhizosphere microbiota composition of dwarfing rootstock under control and drought conditions. To start the experiment, we need to confirm whether apple rootstocks release 4-MU and whether there is an increased 4-MU in root exudates under drought condition, we performed HPLC–MS analysis using root exudates. As shown in Supplemental Figure 8, a significant increase of 4-MU in response to drought stress was observed in vigorous rootstock. Then, we performed the bacterial and fungal ribosomal marker gene profiling. The high-throughput sequencing yielded a total of 1583 prokaryotic operational taxonomic units (OTUs) (Supplemental Table S24) and 453 fungal OTUs (Supplemental Table S25).

We first investigated the impact of 4-MU inoculation on the bacterial and fungal community of dwarfing rootstock rhizosphere under control and drought conditions. Among the comparisons of Chao1 and Shannon diversity for 4-MU treatment under control and drought conditions, only the bacterial communities in rhizosphere in the 4-MU and drought treated plants (D_125) showed lower Chao1 diversity compared to that in non-treated plants under drought stress condition (D_0) (*p* = 0.032, method = Wilcoxon) (Supplemental Figures 9A and B). Notably, the Partial Canonical analysis of Principal Coordinates (CAP) revealed a clear separation of bacteria between control and drought treatment groups and between 4-MU treated and untreated groups (Variance = 33.6%, *p* = 0.001) (Supplemental Figure 9C). Additionally, the CAP analysis also showed a clear separation of fungi between 4-MU treated and untreated samples in the drought environment, while a slight separation in the control environment (Variance = 29.4%, *p* = 0.001) (Supplemental Figure 9D).

**Figure 7:**
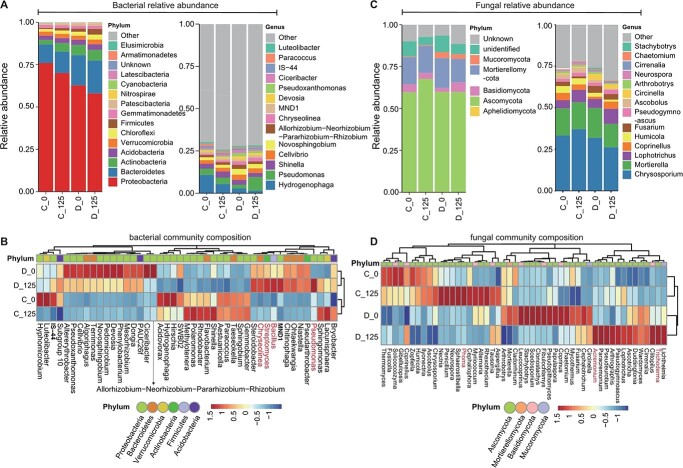
The impact of 4-MU on the bacterial and fungal community in rhizosphere of dwarfing rootstock under control and drought conditions. (A) Bar charts showing the relative abundance of bacterial phyla and genera detected across four samples. (B) Heat map of the bacterial community composition with cluster analysis among the top 45 bacterial genera across samples. (C) Bar charts showing the relative abundance of fungal phyla and genera detected across four samples. (D) Heat map of the fungal community composition with cluster analysis among the top 50 fungal genera across samples. C_0: M9-T337 under control condition, C_125: M9-T337 under 125 μM 4-MU treatment, D_0: M9-T337 under drought treatment, D_125: M9-T337 under 125 μM 4-MU and drought treatment. M9-T337 represents dwarfing rootstock.

As shown in Figure 7A, members of *Proteobacteria* were the dominant bacterial phylum across all samples, followed in order by *Bacteroidetes*, *Actinobacteria*, *Acidobacteria*, *Verrucomicrobia*, *Chloroflexi*, *Firmicutes* and *Gemmatimonadetes.* Compared to rhizosphere without 4-MU treatment in phylum level, the abundance of *Actinobacteria* and *Firmicutes* increased and the abundance of *Proteobacteria* decreased significantly in the rhizosphere treated with 4-MU under control or drought conditions. *Hydrogenophaga*, *Pseudomonas*, *Shinella, Cellvibrio Novosphingobium*, *Allorhizobium-Neorhizobium-Pararhizobium-Rhizobium*, and *Chryseolinea* accounted for relatively high genera in all samples (Figure 7A). The heat map of the top 45 bacterial genera indicated that 4-MU inoculation accumulated some common beneficial genera abundance (e.g. *Pseudomonas*, *Bacillus*, *Streptomyces*, and *Chryseolinea*) in the rhizosphere under drought condition, while 4-MU inoculation significantly decreased the relative abundance of *Shinella*, *Flavobacterium*, *Rhodobacter*, *Polaromonas*, and *Methylotenera* under well-watered conditions (Figure 7B). Consistent with the results in Figure 7B, the common beneficial *Chryseolinea*, *Bacillus*, and *Streptomyces* were the dominant biomarkers under drought stress condition and *Flavobacterium* and *Rhodobacter* were the dominant biomarkers under well-watered condition (Supplemental Figure 10). As for fungal community, *Ascomycota*, *Mortierellomycota*, *Basidiomycota* and *Mucoromycota* were the most dominant phylum among all the samples. At the genus level, *Chrysosporium*, *Mortierella*, and *Lophotrichus* dominated all samples (Figure 7C). Compared to rhizosphere without 4-MU treatment in genera level, inoculation of 4-MU significantly promoted the relative abundance of beneficial *Acremonium*, *Trichoderma*, and *Phoma* (Figure 7C and D).

In order to know specific microbes in response to 4-MU inoculation under control and drought conditions, we used DESeq2 to compare the relative abundance of individual OTUs. Compared to 4-MU untreated rhizosphere, we detected 18 enriched and 54 depleted bacterial OTUs, along with 15 enriched and 15 depleted fungal OTUs in the rhizosphere treated with 4-MU under well-watered conditions. Meanwhile, 75 enriched and 20 depleted bacterial OTUs were identified in 4-MU treated rhizosphere samples, as well as 34 enriched and 17 depleted fungal OTUs compared to 4-MU untreated rhizosphere samples under drought stress condition (Supplemental Figure 11). These results showed that 4-MU inoculation might affect both the bacterial and fungal community in rhizosphere, especially under drought stress condition.

### Exogenous 4-MU enhances drought tolerance of dwarfing rootstock either directly or indirectly.

**Figure 8:**
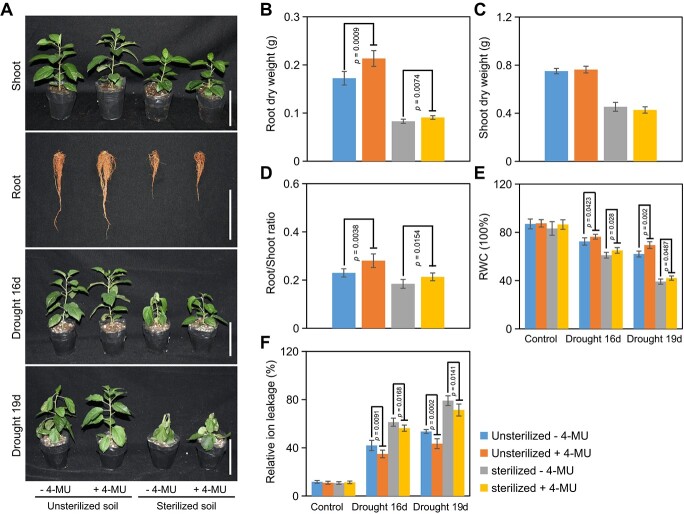
4-MU enhances the drought tolerance of dwarfing rootstock either directly or indirectly. (A) The impact of microorganism on drought tolerance of 4-MU to dwarfing rootstock. (B–D) Shoot dry weight (B), Root dry weight (C), and Root/shoot ratio (D) under control condition. (E) The relative water content (RWC) of leaves under drought stress in both unsterilized and sterilized soil. (F) The relative ion leakage of leaves under drought stress in both unsterilized and sterilized soil. M9-T337 represents dwarfing rootstock. Error bars indicate standard deviation (n = 6). *p* values from Student’s two-tailed *t*-test.

To determine whether the increased drought tolerance in dwarfing rootstock due to 4-MU is a result of its effects on the host plants or through direct regulation of microbiome composition, we assessed the impact of microorganisms on the drought tolerance of dwarfing rootstock treated with 4-MU. It was obvious that the growth of plants was impacted by sterilized soil, whereas 4-MU enhanced root growth but did not affect aerial parts, regardless of soil sterilization (Figures 8A-D). After 4-MU treatment, the root dry weight significantly increased by 23% in unsterilized soil, while in sterilized soil, it increased by 10% (Figure 8B). Consistently, the increase of root-to-shoot ratio was higher in 4-MU-treated plants in unsterilized soil than that in sterilized soil (Figure 8D). Taken together, all the results strongly suggest that the increased drought tolerance observed in dwarfing rootstock may be attributed not only to the direct impact of 4-MU on the host plants, but also its capacity to attract beneficial microbes that boost drought tolerance. We also measured the RWC and relative ion leakage of leaves in response to drought stress both in unsterilized and sterilized soils. 4-MU increased RWC and decreased relative ion leakage in response to drought stress in both unsterilized and sterilized soils (Figures 8E and F).

### Discussion

Under the background of climate change, drought stress is becoming a more and more serious threat to apple production. Therefore, it is necessary to identify determinants that can improve the drought tolerance of apple [3]. In present study, we used transcriptomic and metabolomic analyses to investigate the difference in drought tolerance of two apple rootstocks and identified that 4-MU was significantly induced in the roots of vigorous rootstock under drought stress condition. Exogenous 4-MU inoculation facilitated drought tolerance of dwarfing rootstock, in terms of root-to-shoot ratio, leaf relative water content, photosynthetic parameters, and WUE.

To cope with drought stress, plants have evolved a series of strategies at morphological, physiological, biochemical, and molecular levels, including osmotic adjustment. Osmoregulation under drought stress in plants depends on synthesis and accumulation of osmoprotectants or osmolytes [49]. For instance, increased sugars and sugar alcohols by drought stress can significantly reduce the cell osmotic potential and play a critical role in osmotic adjustment [50]. Numerous studies have demonstrated that plants typically convert starch to sugars or produce metabolites that serve as osmoprotectants to mitigate the adverse effects of drought stress [51]. Moreover, studies have revealed that plants under drought stress accumulated amino acids, indicating their potential roles in the osmotic adjustment that affects plant yield and development [49]. Based on the co-upregulated DEGs by drought, the vigorous and dwarfing rootstocks shared common biological processes under drought condition, particularly the osmotic adjustment, illustrating the common tolerant mechanism of these two rootstocks to drought stress. The common biological processes, including the metabolism of starch, sucrose, galactose, arginine and proline under drought stress, were further supported by the co-upregulation of DRMs (such as trehalose, sorbitol, lactitol, threonine, aspartate, asparagine, arginine, and proline) detected both in vigorous and dwarfing rootstocks. Abiotic stress tolerance, including drought stress, has been demonstrated to be influenced by a number of phytohormones [52, 53]. Interestingly, the down-regulated DEGs both in vigorous and dwarfing rootstocks under drought stress were significantly enriched in the plant hormone signal transduction pathways, indicating the potential involvement of plant hormones in drought response of both rootstocks. In addition, KEGG analysis illustrated that 16 up-regulated DEGs in the roots of drought-treated groups were involved in the plant hormone signal transduction, such as abscisic acid, auxin, cytokinin, jasmonic acid. This further highlights the important functions of plant hormones in regulating how the two rootstocks responds to the drought stress.

According to previous studies, one key method by which plants deal with stressful environments is the rapid perception and activation of secondary metabolites [52, 54]. In addition to serving as stress mediators, root secondary metabolites such as organic acids, phenols, flavonoids, and terpenoids also serve as ecosystem response catalysts to drought stress [55]. Consistent with the previous findings, our findings demonstrated that some secondary metabolites, including organic acids, lipids, benzenoids, phenylpropanoids, and organooxygen compounds, were up- or down-regulated in response to drought stress both in vigorous and dwarfing rootstocks, indicating a genotype-specific adaption mechanism to cope with drought stress. At the metabolites scale, less DRMs were detected in vigorous rootstock in response to drought treatment, indicating a stable metabolome in response to drought treatment. This is consistent with a smaller change in metabolites in drought-tolerant varieties than the drought-intolerant varieties in sesame [56], *Carthamus tinctorius* L. [57], and rice [58]. Meanwhile, we detected the difference in accumulated metabolites between vigorous and dwarfing rootstocks and found that the most up-regulated DRMs in vigorous rootstock were involved in organic acids such as oxopentanoic acid, dihydroxybutyric acid, malic acid, threonic acid, methylglutaric acid, ursolic acid, corosolic acid, liquoric acid, and docosapentaenoic acid. Previous studies demonstrated that organic acids are essential for enhancing plants' ability to adapt to drought stress [54, 59]. As a storage metabolite of the ascorbate metabolism pathway, threonic acid is elevated to sustain cellular integrity and stability [39]. A previous study in rice also indicated that ursolic acid could promote stress tolerance by triggering nitric oxide production and limiting toxic ion as well as ROS accumulation [41]. Notably, threonic acid and ursolic acid were accumulated greatly in vigorous rootstock under drought condition, while obviously reduced in dwarfing rootstock, similar to the previous finding, in which the bean sensitive genotypes exposed to osmotic stress exhibited reduced levels of organic acids [60]. Similarly, the accumulation of malic acid in two wheat genotypes maintained intracellular ionic balance and nutrient uptake to mitigate drought stress [61]. In this study, the malic acid levels significantly increased in vigorous rootstock in response to drought stress, but did not alter significantly in dwarfing rootstock. Nasrollahi et al. (2014) indicated that intense or periodic drought stress increased liquoric acid content in liquorice plants [62]. Furthermore, liquoric acid has the ability of ROS scavenging, resulting in a distinct reduction in the oxidative damage [63]. Our results showed that the drought treatment accumulated liquoric acid in the roots of vigorous rootstock, but not in dwarfing rootstock. All these observations suggested that organic acids may contribute to the drought tolerance in vigorous rootstock. However, the exact role of oxopentanoic acid, dihydroxybutyric acid, methylglutaric acid, corosolic acid, and docosapentaenoic acid in drought resistance is still uncertain, that require further investigation.

A study by Li et al. (2011) reported that 4-MU (active aglycone forms) accumulated in roots, where it is partially transformed by UGTs to 4-MUG (inactive glycoside forms) [44]. It has been shown that coumarin glycosides are produced with its related aglycons (the bioactive form) as the plant’s rapid response to pathogen attack [64] and abiotic stress [13, 65]. The same thing was found in our study, as evidenced by changes of 4-MU and 4-MUG in drought-treated vigorous rootstock, as compared to the well-watered vigorous rootstock. Hence, accumulation of 4-MU and UGTs-mediated glycosylation of 4-MU may represent an adaptative mechanism to respond to drought stress in vigorous rootstock.

Many studies suggested that the application of exogenous couriman is of great significance for alleviating abiotic stress in plants. Previous reports indicated that coumarin improved plant tolerance to salinity by enhancing glyoxalase system, ion homeostasis, osmoregulation process, and antioxidant defense system [16, 66]. Li et al. (2011) demonstrated that 4-MU participates in root branching partly via auxin redistribution in *Arabidopsis* [44]*.* Given the tight connection between plant root architecture and drought tolerance, involvement of 4-MU in plant drought stress response is very possible and reasonable. Similarly, our results indicated that exogenous application of 4-MU on dwarfing rootstock enhanced the root development, meanwhile the aboveground was comparable under well-water condition. Furthermore, we discovered that 4-MU inoculation significantly reduced the plant growth inhibition, leading to a larger root-to-shoot ratio under drought stress. Lastly, exogenous application of 4-MU increased Gs, Ci, and WUE under drought stress, indicating that 4-MU promoted photosynthesis by reducing stomatal limitation. In addition, 4-MU regulated drought responsive genes that are mainly involved in root development, cell cycle, auxin transport, and response to auxin stimulus, which is consistent with the previous findings that 4-MU-induced root branching due to auxin distribution [44]. Taken together, 4-MU increased dwarfing rootstock drought resistance by promoting the root development, reducing stomatal limitation, improving photosynthetic capacity, and increasing WUE.

Drought stress can alter the plant metabolic pathways as well as the specific root exudates, which selectively facilitate plant–microbe interactions in order to relieve the effects of drought stress. Microorganisms associated with plants are important in reducing the effects of drought stress [67]. One strategy to mitigate drought stress in crops is to make full use of root rhizosphere microorganisms, but knowledge about the relationship between plant hosts and their microbiomes during drought remains unclear [68]. The effects of 4-MU treatment on the microbial community suggested that the 4-MU inoculation increased the richness and diversity of microbial community. It has been shown that the application of *Actinobacteria*, *Firmicutes*, and *Chloroflexi* are the most prominent bacterial phylum in response to drought treatment [69, 70], and they can benefit plant host fitness under drought stress condition [71, 72]. Interestingly, the 4-MU inoculation significantly promoted the increase in the relative abundance of bacterial phylum *Actinobacteria*, *Firmicutes*, and *Chloroflexi*, implying the significant roles of 4-MU in drought resistance in apple rootstock via reshaping bacterial communities in rhizosphere. We also observed that the *proteobacteria* relative abundance was significantly decreased by drought treatment, consistent with previous reports [73, 74]. Under drought condition, at genus level the *Pseudomonas*, *Bacillus*, *Streptomyces*, and *Chryseolinea* were highly enriched after 4-MU treatment in the rhizosphere. Many strains in these genera could promote the plant growth and improved plants resistant to drought stress. Previous studies have demonstrated that *Pseudomonas* and *Bacillus* could alter the root system development by changing the volatile and secondary metabolites, as well as affecting the hormone homeostasis or signaling [75–77]. The root colonization by *Pseudomonas* increased the water stress tolerance of plants by producing osmoreceptors, photosynthetic pigments, or abscisic acid that led to an improved WUE and biomass accumulation [30, 78]. Furthermore, *Bacillus*-inoculated plants showed physiological responses under drought stress including the higher root/shoot ratio that could mitigate drought stress [79, 80]. In addition, Abbasi et al. (2020) suggested that *Streptomyces* has alleviated drought stress by increasing proline and total sugar contents in the inoculated tomatoes [32]. All these observations revealed that the 4-MU driven selection of putatively beneficial bacteria such as *Pseudomonas*, *Bacillus*, and *Streptomyces* in response to drought stress would be of great significance to the drought resistance of dwarfing rootstock. Meanwhile, in present study the fungal genus *Acremonium*, *Trichoderma*, and *Phoma*, which were previously reported to facilitate drought tolerance of plants through osmotic adjustment, ROS scavenging, production of plant hormones, mineral solubilization, enhanced nutrient uptake, and improved photosynthesis [33, 35, 81, 82], were also found to be increased in relative abundance in the 4-MU treated rhizospheres under well-watered or drought conditions. In addition, we also observed a greater increase in root-to-shoot ratio in 4-MU-treated plants grown in unsterilized soil as compared to sterilized soil. All these observations indicated that 4-MU driven beneficial bacteria or fungi might be a significant player in drought resistance in perennial apple trees.

## Materials and methods

### Plant materials, growth conditions, and drought treatment

All the field experiments in this work were carried out at Wuquan Experimental Station, Yangling City, Shaanxi Province, China. For transcriptomic and metabolomic analysis, in June 2020, shoot segments of *M. sieversii* and M9-T337 were rooted on 1/2 MS media and then transplanted to the pots (30 cm × 18 cm) and grown for additional 30 days. After that, we randomly divided the plants into two groups based on their sizes (Control group and Short-term drought treatment group). TDR (FS6430, American Aurora Spectrum Technology Co., Ltd.) was used to determine soil volumetric water content (VWC) and a weighing method was used to determine soil mass water content (MWC). For the short-term drought treatment, the soil VWC and MWC of control group was maintained at 45% and 80%, respectively. Then, water was withheld from plants of short-term drought treatment group for 20 d until VWC and MWC reached 5% and 20%, respectively. The root samples were collected for RNA (three biological replicates) and metabolite extraction (six biological replicates).

To assess the roles of 4-MU under long-term drought stress, in June 2021, the *M. sieversii* and M9-T337 seedlings were transplanted to the pots and grown for an additional 30 d. After that, we randomly divided the plants into six groups based on their sizes (Control group treated with 0, 125, and 500 μM 4-MU and long-term drought treatment group treated with 0, 125, and 500 μM 4-MU). Each group has 15 plants. The VWC and MWC of the control group were maintained at 43–48% and 70–85%, respectively. For the long-term drought treatment, daily irrigation maintained VWC at 18–23% and MWC at 45–55%, respectively. All of the treatments lasted for two months.

To study the effect of microorganisms on the drought tolerance of 4-MU to dwarfing rootstock, M9-T337 seedlings were transplanted into soil and grown for an additional 30 d. Four treatments were tested: (1) unsterilized soil without 4-MU, (2) unsterilized soil with 125 μM 4-MU, (3) sterilized soil without 4-MU, and (4) sterilized soil with 125 μM 4-MU. Afterward, water was withheld for 19 days, and drought resistance indicators were measured at 16th and 19th days.

For the root exudate detection, *M. sieversii* seedlings were transplanted into soil and grown for an additional 30 d. Plants with similar size were chosen for hydroponic cultivation with a 1/2 Hoagland’s solution. To allow the plants to adapt to a hydroponic condition, the plants were pre-cultured for about 10 days before being divided into two groups. The nutrient solution was refreshed every 3 days with an adjusted pH 5.8. After pre-culturing, one group of plants was transferred to grow in 1/2 Hoagland’s nutrient solution containing 10% PEG6000 for 24 hours. The solution (three biological replicates) was then concentrated using a rotary evaporator (BVCHI, Switzerland) and finally detected by HPLC–MS (AGLIENT, USA).

### RNA-sequencing analysis

As recommended by the manufacturer, we extracted total RNA from the roots using RNAprep Pure Plant Kit (TIANGEN, China). Three biological replicates were used for RNA-sequencing. RNA-sequence was performed as described by Owens et al [83]. Briefly, the sequencing libraries were generated. Then, we sequenced them on an Illumina Novaseq 6000 platform. After quality control and filtration, we used HISAT2 v2.0.4 to map clean reads to the apple reference genome. The screening criteria: |log2FC (fold change)| ≥ 1 and *p*-value ≤0.05. KEGG (Kyoto Encyclopedia of Genes and Genomes) and GO (Gene Ontology) analysis were performed by GOseq and KOBAS softwares, respectively.

### Metabolite extraction and LC–MS analysis

As described with some modifications (De Vos et al) [84], metabolite extraction and LC–MS analysis were conducted. The root samples (six biological replicates) were ground into fine powder which (100 mg) was then resuspended in pre-cooled 50% methanol buffer (120 μL), followed by mixing with vortex for 1 min and incubate for 10 min at room temperature, then overnight at −20°C. After centrifugation, the supernatants were transferred to 96-well plates and stored at −80°C before LC–MS analysis.

### Metabolomics data processing

The raw data files from the LC–MS were processed using the toolbox in R software, including XCMS, CAMERA and metaX. Based on the complete information (retention time and m/z pairs) of each peak and in-house or public database, metabolites were identified. To detect outliers and batch effects, the pre-processed dataset was subjected to principal component analysis (PCA). In addition, we employed partial least square discriminant analysis (PLS-DA) to maximize and recognize the variables carrying the class-separating information. Variable importance in projection (VIP) measures the importance of variables in the model, based on the weighted sum of squares of the PLS-DA analysis. The screening criteria: fold change ≥2 or fold change ≤0.5; *p* ≤ 0.05 and VIP ≥ 1.

### Physiological responses to drought treatment

To measure the leaf relative water content, we sampled the second and third fully expanded leaves from the top and weighed them as fresh weight, then weighed them again as saturated weight after soaking them in water for 12 hours. Lastly, the leaves were transferred to an oven until reach to a constant (dry) weight. Calculation of relative water content:

\begin{align*} \mathrm{Leaf}\ &\mathrm{relative}\ \mathrm{water}\ \mathrm{content}\\&=\frac{\mathrm{Fresh}\ \mathrm{weight}-\mathrm{Dry}\ \mathrm{weight}\ }{\mathrm{Saturated}\ \mathrm{weight}-\mathrm{Dry}\ \mathrm{weight}} \times 100 \end{align*}

For ion leakage assay, water was withheld from plants for 16 and 19 days, then the leaves were punched into leaf discs. The leaf discs were placed in glass tubes containing 8 mL deionized water for five hours, and electrolyte leakage was measured using a conductivity meter.

Photosynthesis related parameters were monitored using a LI-6400XT portable photosynthesis system (LI-COR, USA). The instantaneous water use efficiency (iWUE) was calculated as follows: iWUE = Photosynthetic rate/Transpiration rate.

Carbon isotopic composition (δ^13^C) analysis were conducted as described by Wang et al. [48]. Briefly, the dried leaves were ground into fine powder. Next, we used an isotope ratio mass spectrometer (Thermo Fisher Scientific) to measure the δ^13^C value. δ^13^C of each sample was calculated as follows:δ^13^C (‰) = [(R_VPDB_/R_sample_)-1]*1000, R_VPDB_ and R_sample_ were the values of ^13^C/^12^C for the international standard VPDB (Vienna Peedee Belemnite) and sample, respectively.

### Microbiome sample collection, library preparation and sequence analysis

In September 2021, rhizosphere samples (five biological replicates) of 4-MU treated and untreated “M9-T337” grown in Wuquan experimental station under control and drought conditions were collected for 16S and Internal Transcribed Spacer (ITS) ribosomal RNA (rRNA) sequencing. Sample preparation, DNA extraction, amplification, and sequencing were conducted as described in an earlier report with slight modifications [85].

Briefly, we first scooped out the plants from their pots and gently shaken them to remove loose soil. The rhizosphere soil samples were placed in polythene bags and immediately transported to the lab on dry ice. Subsequently, fine roots (approximately 1 mm diameter) were collected into a 50 mL falcon tube filled with 30 mL Phosphate buffer solution (PBS). Rhizosphere samples were collected by washing the fine roots in fresh PBS and centrifuging them three times (3000–5000 rpm) for 10 min. The soil washed off from the fine roots was termed the rhizosphere compartment and was used for further sequencing. All the soil samples were stored at −80°C (about one week) before DNA isolation.

DNA was extracted using CTAB method and 16S rRNA/ITS genes were amplified with specific primers for sequencing on Illumina novaseq platform. Clustering of sequences into Operational Taxonomic Units (OTUs) was performed using usearch61 algorithm in QIIME. Rare OTUs (<0.005%) were removed, and only those assigned to Kingdom Bacteria and Fungi were kept. Differential relative abundance analysis was conducted using DESeq2 package. Alpha diversity indices (Chao1 and Shannon) were calculated and differences in community structures were tested for significance with wilcox.test. Beta diversity was evaluated using partial Canonical analysis of Principal Coordinates (CAP) and Bray Curtis metrics. Differentially abundant taxa were identified using linear discriminant analysis Effect Size.

## Supplementary Material

Web_Material_uhad099

## Data Availability

The sequencing data have been deposited to the NCBI with the dataset identifier PRJNA861726.
