# Supplementary material for: 4-methylumbelliferone (4-MU) enhances drought tolerance of apple by regulating rhizosphere microbial diversity and root architecture
Source: Hortic Res. 2023 May 19;10(7):uhad099. doi: 10.1093/hr/uhad099 (PMC10327542; doi:10.1093/hr/uhad099)
Supplement: Web_Material_uhad099 [file web_material_uhad099.zip › supplemental figures.pdf]

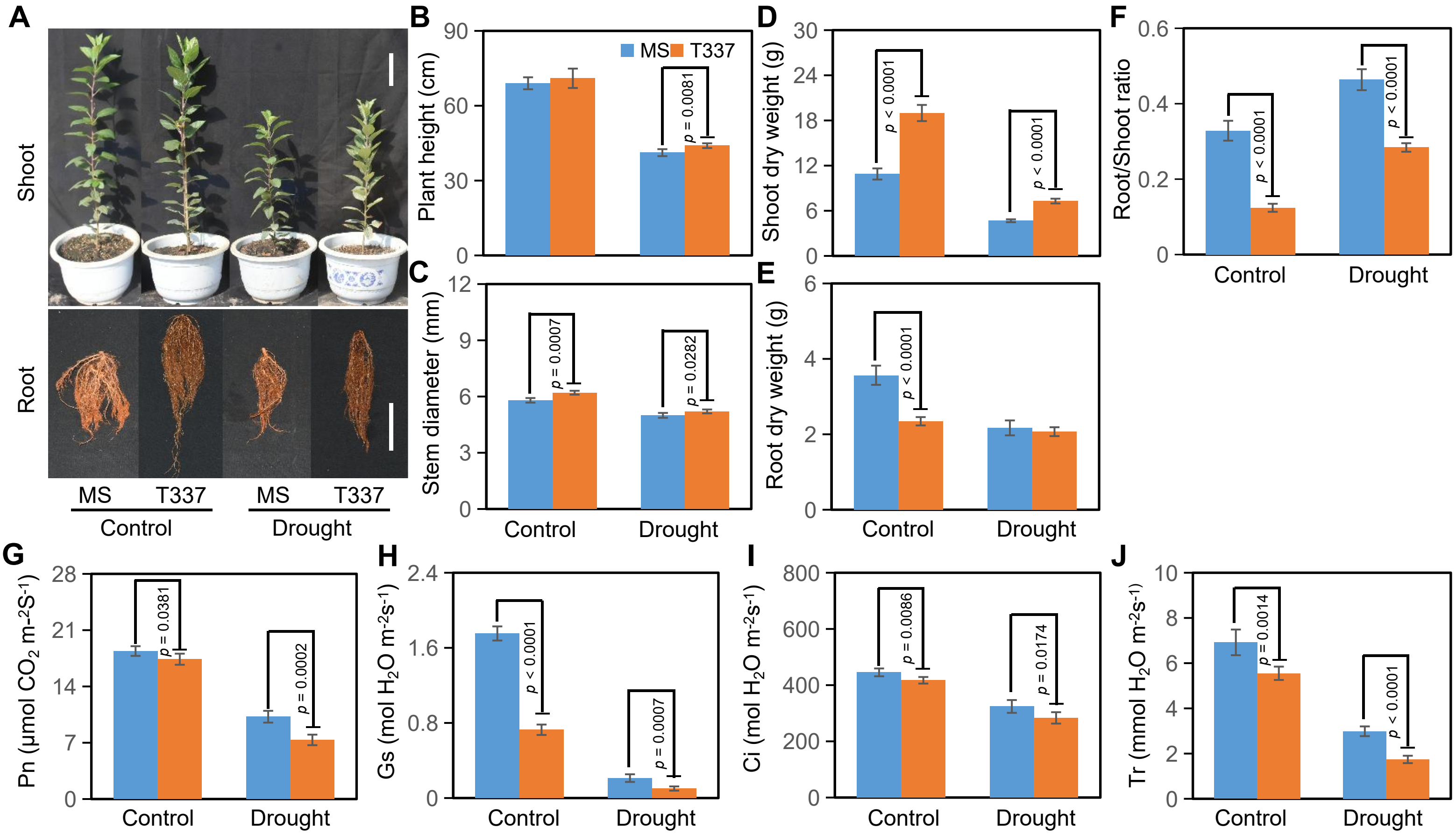

Supplemental figure S1. *M. sieversii* is more tolerant to drought than M9-T337. (A) Morphology of *M. sieversii* and M9-T337 under control (C_) and drought (D_) stress conditions. Bars = 10 cm. (B) Plant height. (C) Stem diameter. (D) Shoot dry weight. (E) Root dry weight. (F) Root/shoot ratio. (G) Net photosynthesis (Pn). (H) Stomatal conductance (Gs). (I) intercellular CO2 concentration (Ci). (J) Rate of transpiration (Tr). MS, *M. sieversii*; T337, M9-T337. Error bars indicate standard deviation (n=5). *p* values from Student's two-tailed *t*-test.

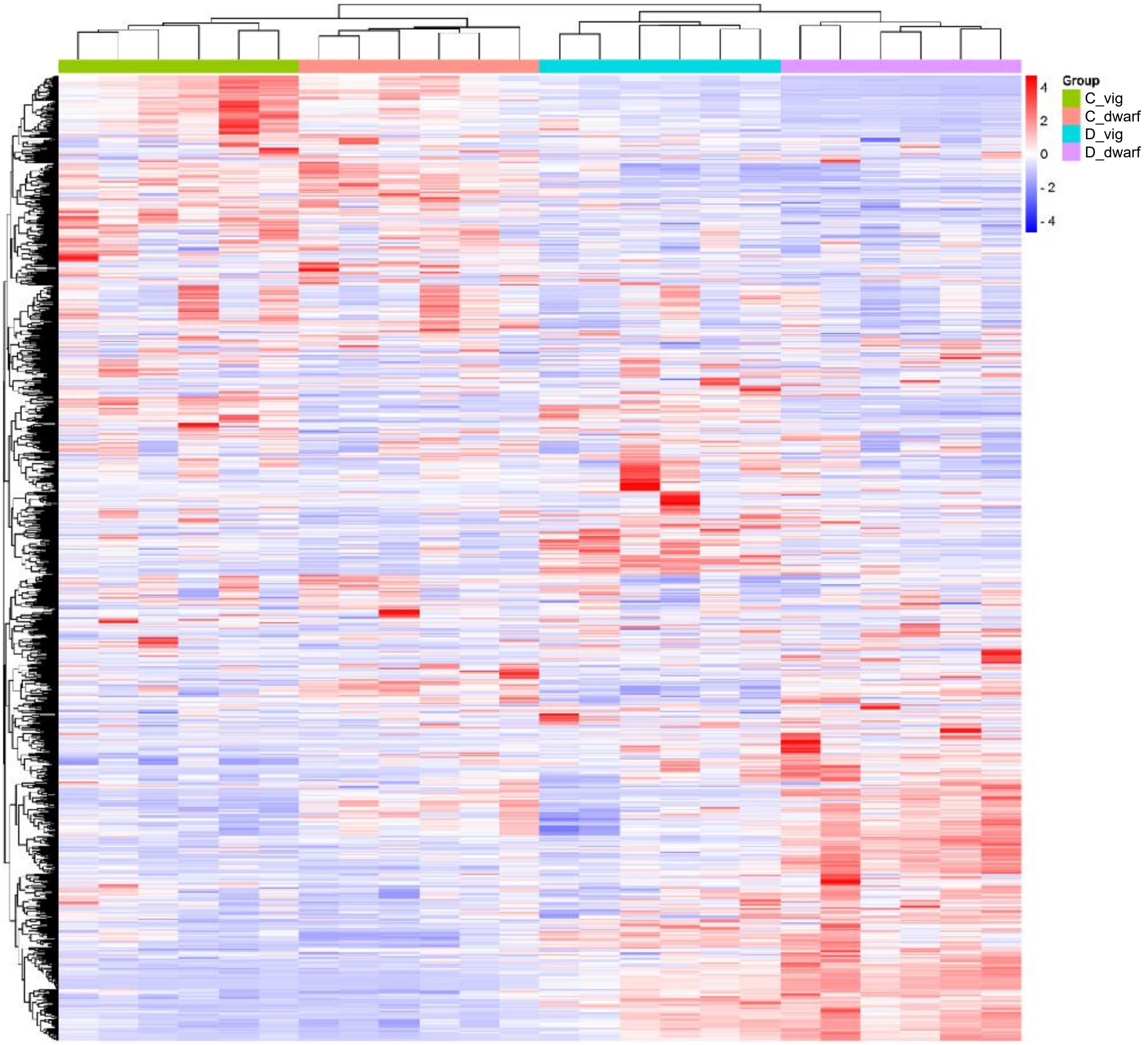

Supplemental figure S2. Identified 953 metabolites both in vigorous and dwarfing rootstocks under control and drought conditions. The colors represent the relative content of the identified metabolites. C_vig: vigorous rootstock under control condition, C_dwarf: dwarfing rootstock under control condition, D_vig: vigorous rootstock under drought treatment, D_dwarf: dwarfing rootstock under drought treatment. *Malus sieversii* and M9-T337 represent vigorous and dwarfing rootstock, respectively.

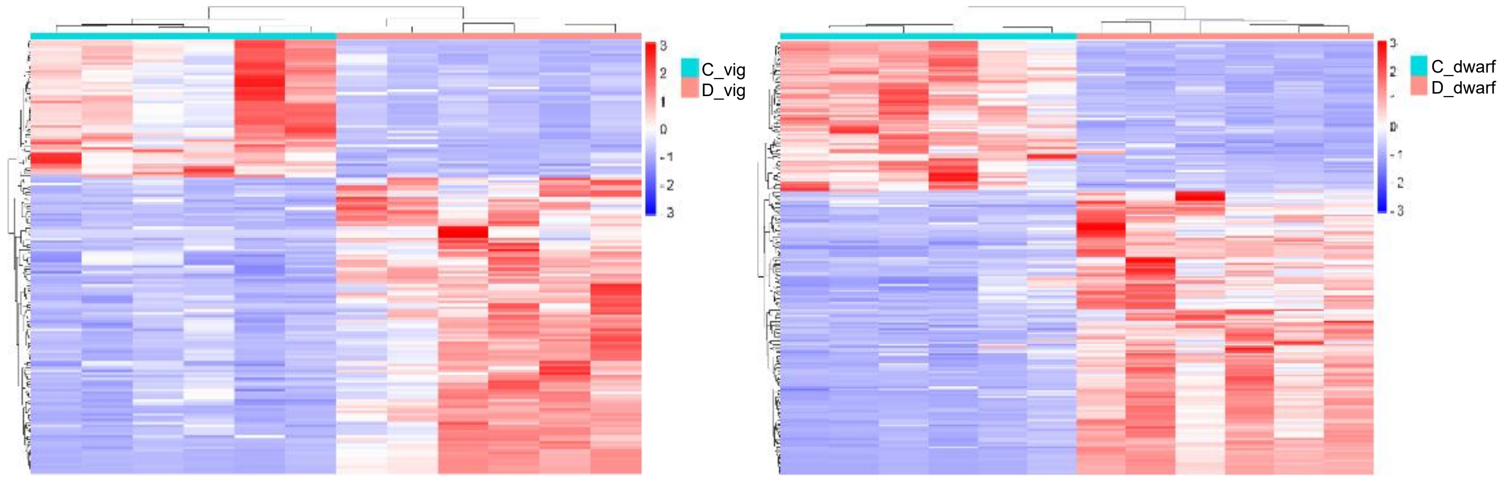

Supplemental figure S3. Heatmaps of identified metabolites in vigorous and dwarfing rootstocks in response to drought stress. The colors represent the relative content of the identified metabolites. C_vig: vigorous rootstock under control condition, D_vig: vigorous rootstock under drought treatment, C_dwarf: dwarfing rootstock under control condition, D_dwarf: dwarfing rootstock under drought treatment. *Malus sieversii* and M9-T337 represent vigorous and dwarfing rootstock, respectively.

ID	Metabolite
neg-M115T52	3-Oxopentanoic acid
neg-M119T52	(S)-3,4-Dihydroxybutyric acid
neg-M131T79_2	L-Asparagine
neg-M133T276	DL-Malic acid
neg-M135T53	Threonic acid
neg-M135T56	Threonic acid
neg-M161T250	3-Hydroxy-3-methylglutaric acid
neg-M287T219	Eriodictyol
neg-M456T217	Amygdalin
neg-M469T389	Ursolic acid
neg-M471T406	Corosolic acid
neg-M483T400	Liquoric acid
neg-M485T398	Ursolic acid
neg-M593T435	LysoPI 18:3; LysoPI 18:3
pos-M119T288	Benzofuran
pos-M147T287	Coumarin
pos-M148T63	DL-Glutamate
pos-M175T286	4-Hydroxynaphthalene-1,2-dione
pos-M177T290	4-Methylumbelliferone
pos-M205T53_2	Eupolauridine
pos-M225T288	gamma-valerolactone
pos-M339T226	4-Methylumbelliferyl-D-glucopyranoside
pos-M339T289	4-Methylumbelliferyl-D-glucopyranoside
pos-M345T329	Docosapentaenoic acid
pos-M353T417	Monolinolenin (9c,12c,15c)
pos-M579T219	Procyanidin B1
pos-M86T89	Piperidine

Supplemental figure S4. The unique and up-regulated metabolites by drought stress in vigorous rootstock. *Malus sieversii* represents vigorous rootstock.

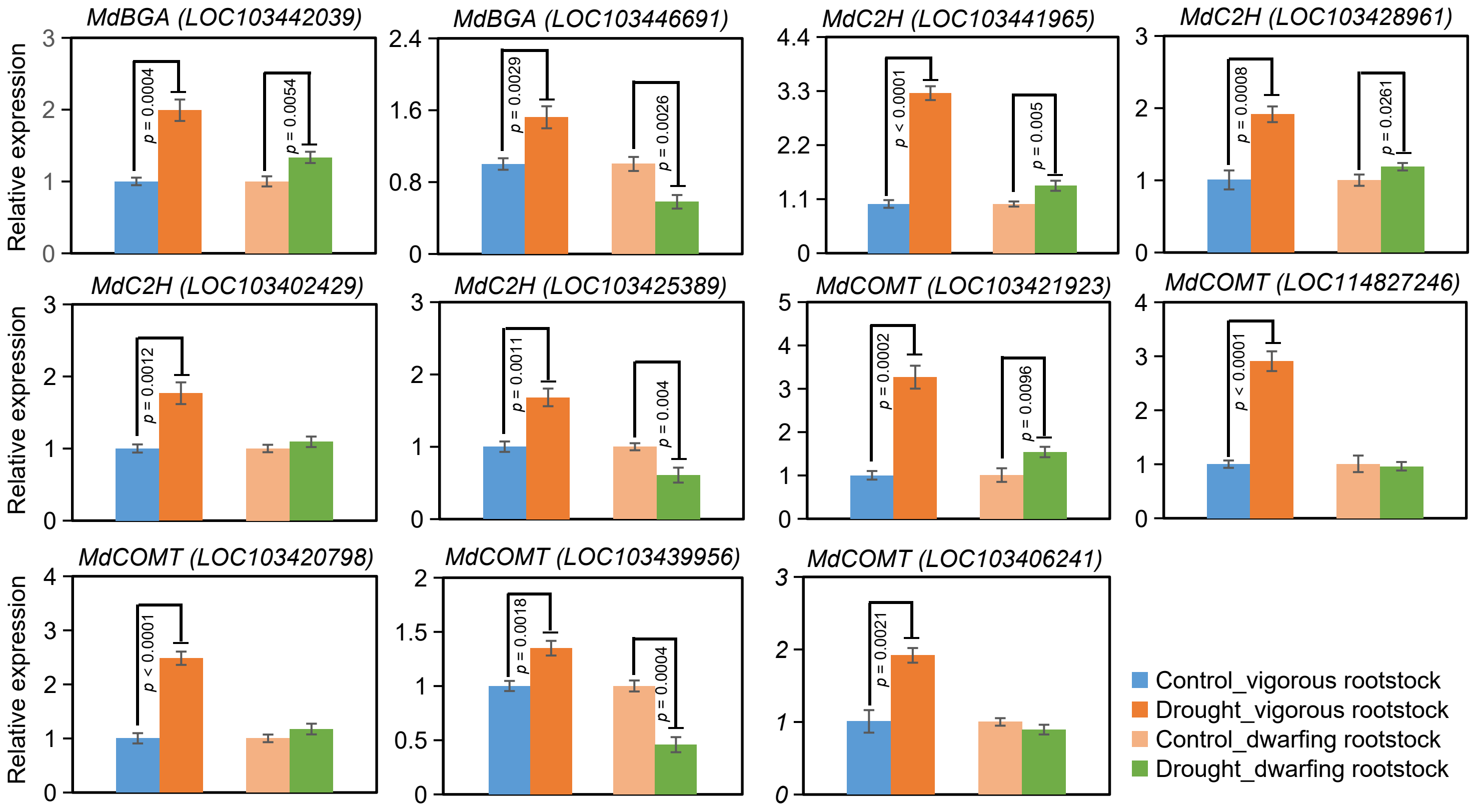

Supplemental figure S5. Relative expression of selected genes related to coumarin biosynthetic pathway in vigorous and dwarfing rootstock in response to drought stress. *Malus sieversii* and M9-T337 represent vigorous and dwarfing rootstock, respectively. *p* values from Student's two-tailed *t*-test.

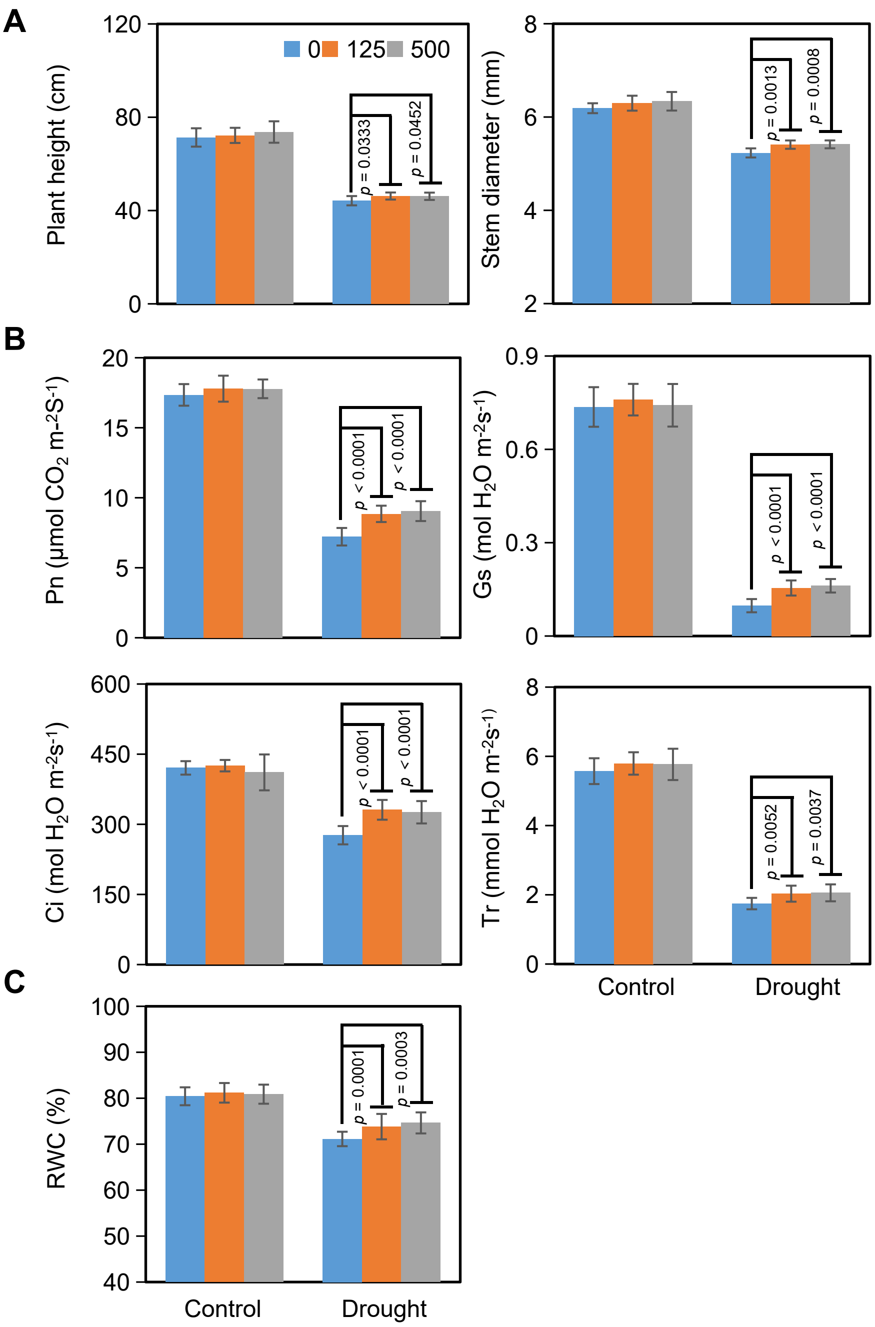

Supplemental figure S6. 4-MU promotes the drought tolerance of dwarfing rootstock. (A) Plant height and stem diameter of plants under control and long-term drought conditions. (B) 4-MU promotes the photosynthesis under long-term drought stress. Pn: photosynthetic rate; Gs: stomatal conductance; Ci: intercellular CO2 concentration; Tr: transpiration rate. (C) 4-MU improved relative water content (RWC) in leaves under drought stress. M9-T337 represents dwarfing rootstock. Error bars indicate standard deviation (n=10). *p* values from Student's two-tailed *t*-test.

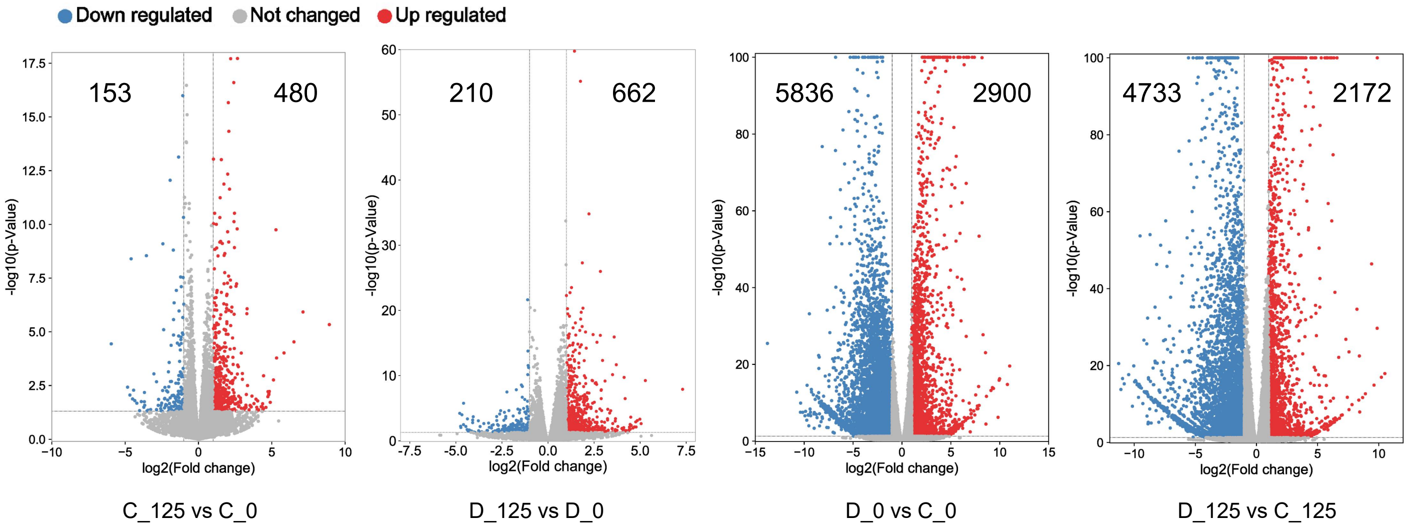

Supplemental figure S7. Volcano plots of DEGs in roots of dwarfing rootstock in response to 4-MU and drought treatment. C_0: M9-T337 under control condition, C_125: M9-T337 under 125 μM 4-MU treatment, D_0: M9-T337 under drought treatment, D_125: M9-T337 under 125 μM 4-MU and drought treatment. M9-T337 represents dwarfing rootstock.

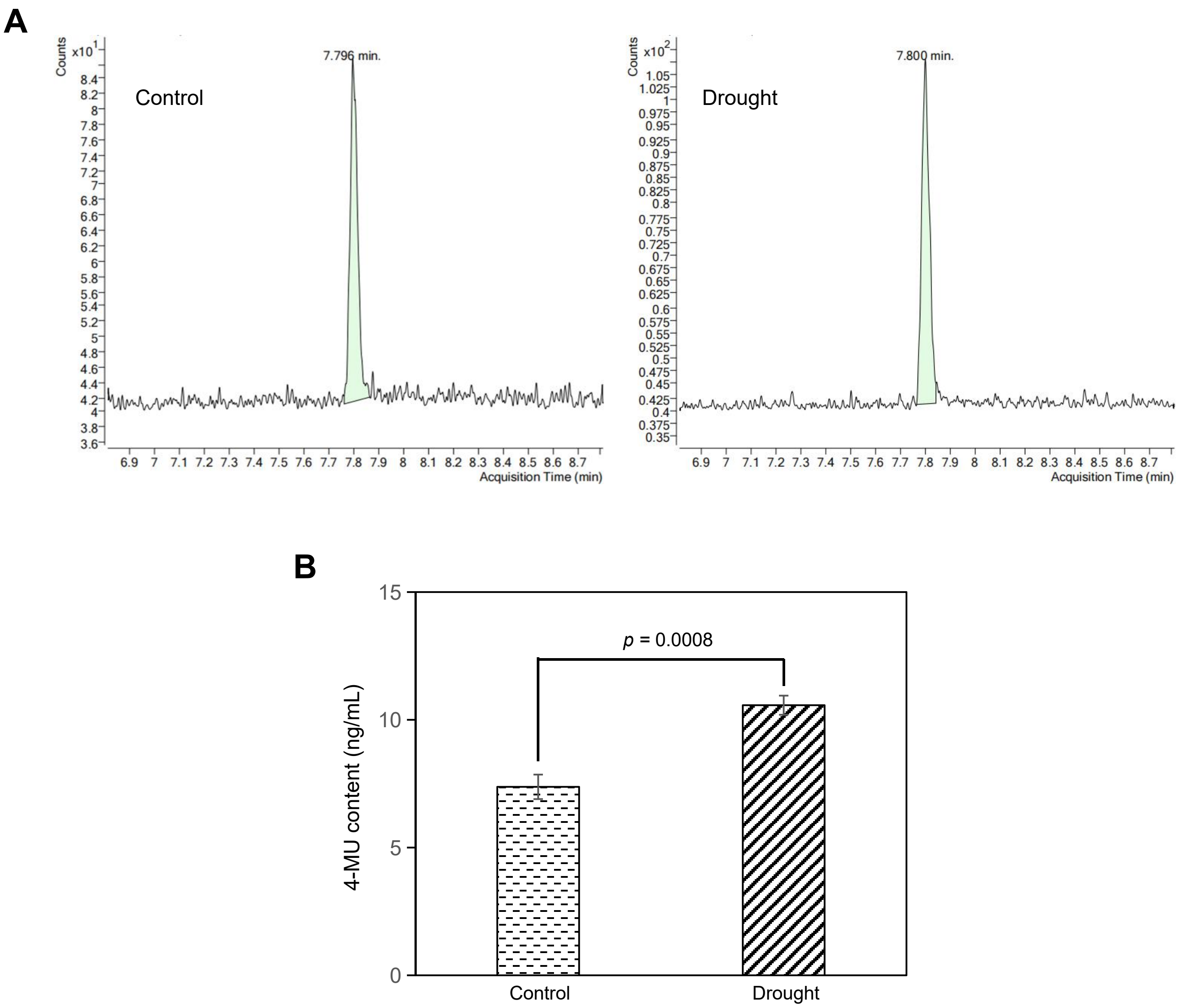

Supplemental figure S8. Drought enhances 4-MU production in root exudates of vigorous rootstock. (A) HPLC-MS profiles of root exudates under control and drought conditions. (B) 4-MU content in root exudates under control and drought conditions. PEG (10%) solution was used to simulate drought stress. *Malus sieversii* represents vigorous rootstock. *p* values from Student's two-tailed *t*-test.

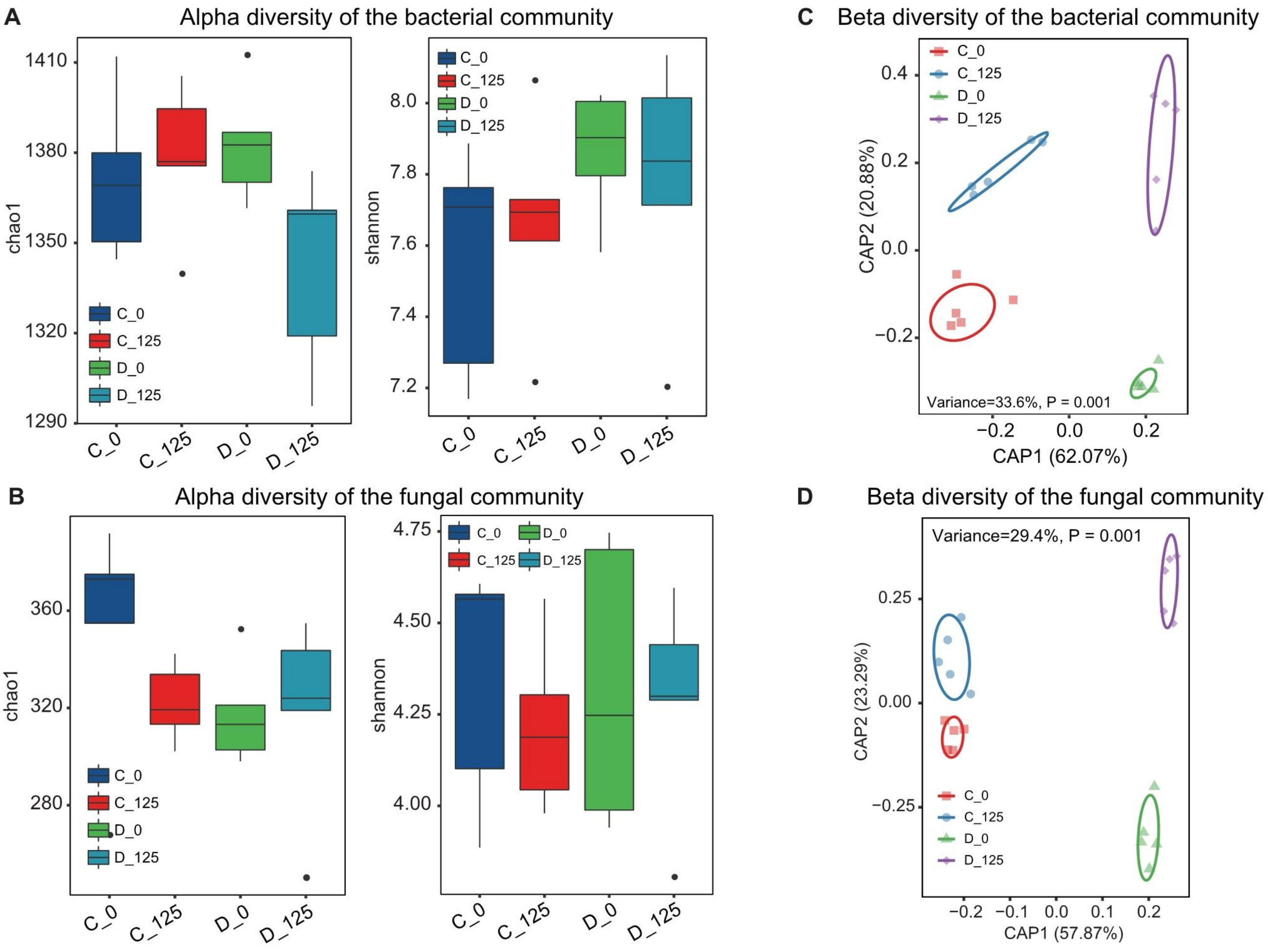

Supplemenntal figure S9. The impact of 4-MU on the alpha and beta diversity of bacterial and fungal community in rhizosphere of dwarfing rootstock under control and drought conditions. (A) Boxplot illustrating the differences in Chao1 and Shannon diversity of the bacterial community. (B) Boxplot illustrating the differences in Chao1 and Shannon diversity of the fungal community. (C) Beta diversity analysis of the bacterial community based on Bray-Curtis distance matrix. (D) Beta diversity analysis of the bacterial community based on Bray-Curtis distance matrix. Samples were compared using Canonical analysis of principal coordinates (CAP) methods. C_0: M9-T337 under control condition, C_125: M9-T337 under 125 μM 4-MU treatment, D_0: M9-T337 under drought treatment, D_125: M9-T337 under 125 μM 4-MU and drought treatment. M9-T337 represents dwarfing rootstock.

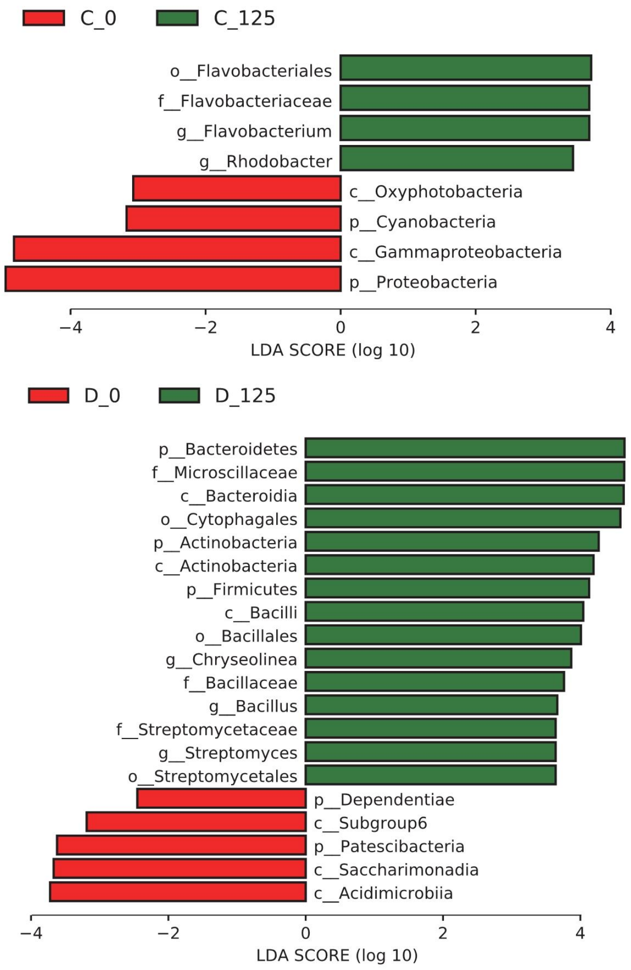

Supplemental figure S10. Linear discriminant analysis Effect Size (LEfSe) estimation of bacterial community. Significance determined using default parameters (Kruskal Wallis test $p < 0.05$ and LDA score > 2). Magnitude of each bar represents the effect size. C_0: M9-T337 under control condition, C_125: M9-T337 under 125 μM 4-MU treatment, D_0: M9-T337 under drought treatment, D_125: M9-T337 under 125 μM 4-MU and drought treatment.

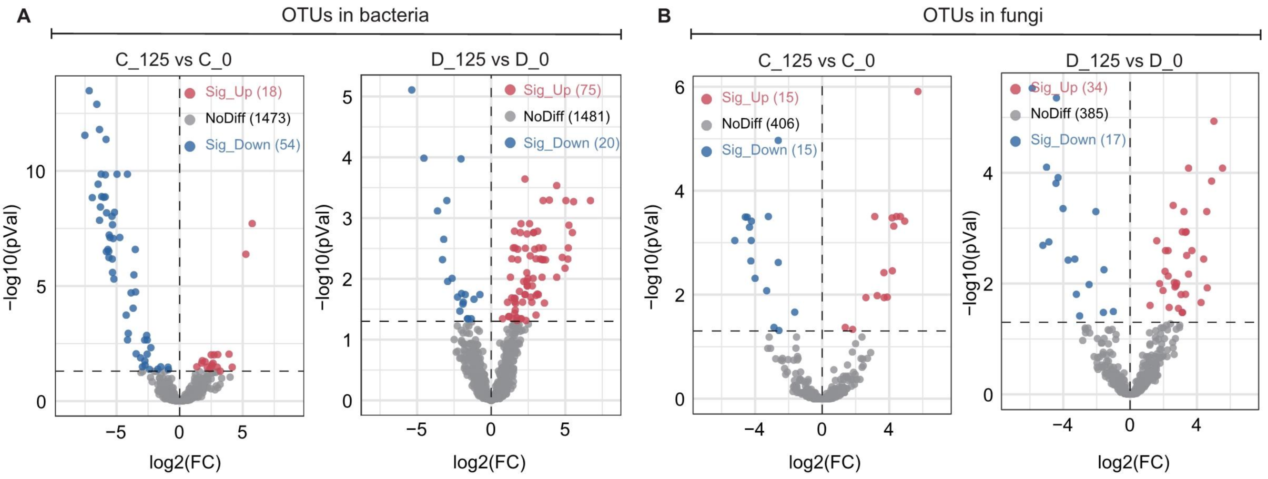

Supplemental figure S11. Volcano plot represents OTUs with significant abundance differences after 4-MU treatment in dwarfing rootstock under the control and drought conditions. C_0: M9-T337 under control condition, C_125: M9-T337 under 125 μM 4-MU treatment, D_0: M9-T337 under drought treatment, D_125: M9-T337 under 125 μM 4-MU and drought treatment. M9-T337 represents dwarfing rootstock.
